# When shapes are more than shapes: perceptual, developmental, and neurophysiological basis for attributions of animacy and theory of mind

**DOI:** 10.3389/fpsyg.2023.1168739

**Published:** 2023-09-07

**Authors:** Sajjad Torabian, Emily D. Grossman

**Affiliations:** Visual Perception and Neuroimaging Lab, Department of Cognitive Sciences, University of California, Irvine, Irvine, CA, United States

**Keywords:** social cognition, cognitive development, animacy, agency, theory of mind, motion perception, Heider and Simmel, default-mode network

## Abstract

Among a variety of entities in their environment, what do humans consider alive or animate and how does this attribution of animacy promote development of more abstract levels of mentalizing? By decontextualizing the environment of bodily features, we review how physical movements give rise to perceived animacy in Heider-Simmel style animations. We discuss the developmental course of how perceived animacy shapes our interpretation of the social world, and specifically discuss when and how children transition from perceiving actions as goal-directed to attributing behaviors to unobservable mental states. This transition from a teleological stance, asserting a goal-oriented interpretation to an agent's actions, to a mentalistic stance allows older children to reason about more complex actions guided by hidden beliefs. The acquisition of these more complex cognitive behaviors happens developmentally at the same time neural systems for social cognition are coming online in young children. We review perceptual, developmental, and neural evidence to identify the joint cognitive and neural changes associated with *when* children begin to mentalize and *how* this ability is instantiated in the brain.

## 1. Introduction

**Figure 1:**
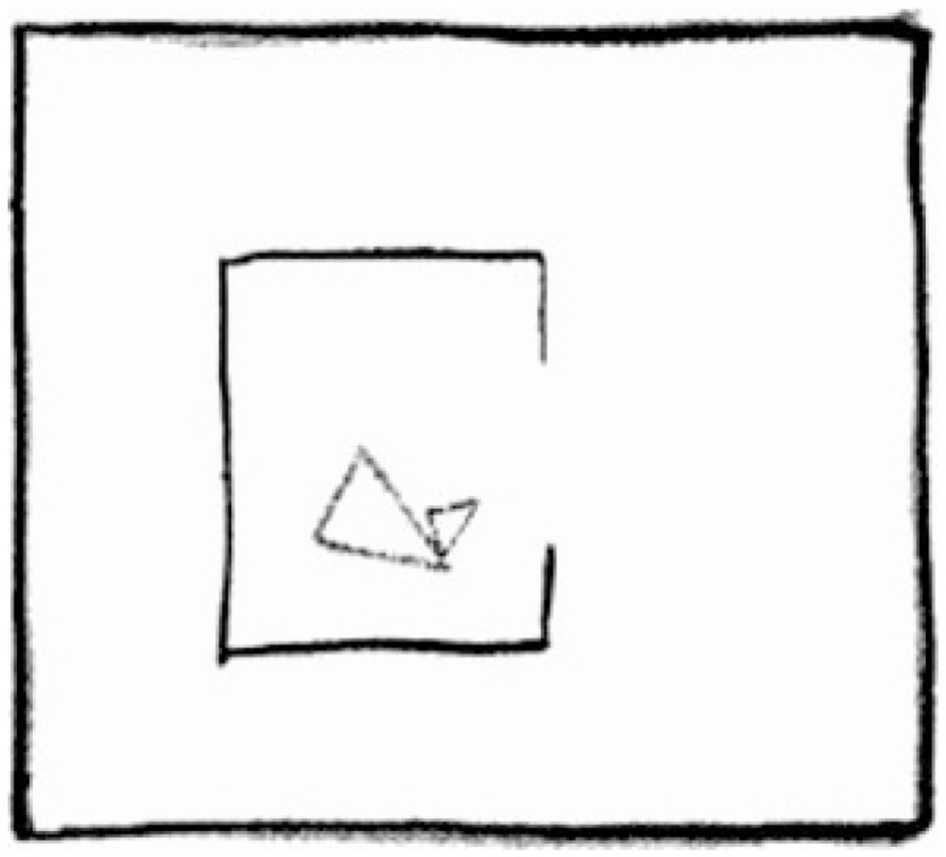
Single frame of Heider-Simmel animation designed by Castelli et al. (2000), depicting a mother who persuades child to go out, conveying theory of mind. Adapted with permission.

In their seminal work on apparent behavior, Heider and Simmel (1944) showed that when humans viewed a two-dimensional animation of simple geometric shapes, their interpretations of the movements tended not toward a physical story. Instead, people perceived the shapes as animated beings and agents, and described their observation in rather abstract terms. For example, when a triangle vibrated in proximity to another triangle, people saw the two as agents who engaged in a social interaction such as fighting. A line of studies followed the work of Heider and Simmel, showing how motion alone can turn objects into living beings. A single frame of an example Heider-Simmel like animation with two interacting shapes is depicted in Figure 1. Recently Ratajska et al. (2020) designed an extended range of social plots to demonstrate that simple shapes of various types, not just triangles and circles, can depict rich narratives beyond conflict interactions, even on a brief timescale (13–23 s compared to 2 1/2 min).

Often cast within the broad framework of theory of mind (ToM, the ability to attribute mental states to others, which are inferred and therefore unobservable, and can be used to make predictions about the behaviors of others, Premack and Woodruff, 1978), the perception of animacy, interactivity and goal directed behaviors derived from Heider-Simmel type animations reflect the human tendency to construct social interpretations and derive inferences about beliefs and desires from movement patterns alone (Baker et al., 2017). In this review, we will discuss perceptual, developmental, and neural underpinnings of perceived animacy and social attributions. Specifically we seek to link the development of neural systems to the ability to draw upon perceptual cues for animacy in order to establish more complex beliefs about the goals of others. We also discuss the evidence that detection of animacy, and to some extent the ability to discern goal-directed behaviors, is not uniquely human. We couch our discussion within the framework that the behavior of animate objects can be broadly categorized into goal-directed and mentalistic (see Gergely and Csibra, 2003, but also Schaafsma et al., 2015 for a systematic deconstruction of theory of mind), motivated by the psychological principle of rational action. This principle states that a bias exists to interpret behaviors as goal-oriented, guided by environmental constraints and mental states, the latter of which will be inferred under the assumption that the agent is performing efficient actions.

To better disentangle goal-directed and mentalistic representations, we review (1) the developmental literature as it offers clear perspectives into how children acquire rich mental representations of the social world around them, as well as (2) findings in monkeys, apes, and chicks. We will discuss neural systems supporting goal-directed and mentalistic representations in adults and the development of those brain systems in children under age two when these cognitive systems come online. In this review we focus on research that employs Heider-Simmel type animations that are deprived of many of the explicit cues that typically signal animacy, the determination that it is appropriate to apply psychological reasoning to a given entity (Csibra et al., 1999), and agency, the capacity to engaged in intrinsically motivated (goal-directed) behavior. This approach is particularly valuable because it is accessible to adults, children and non-humans alike, while also decoupling animacy from the perception of species-specific cues, such as faces and eye gaze. In the following section we first lay the groundwork for studying social behavior, by discussing perceptual cues that give rise to animacy.

## 2. The perceptual determinants of perceived animacy

There are many cues in our environment that signal animacy, intention and goals, including eye gaze, head tilt, facial expressions and body movements (Chang and Troje, 2008). Heider-Simmel type animations are devoid of all of these cues, and nonetheless give rise to the perception of animacy, which lies at the foundation of mental state attributions (Schultz and Frith, 2022). Initiation of movement, change in speed, and change in direction (particularly to avoid a barrier) are all examples of such cues that are readily and reflexively interpreted as signaling animacy (Stewart, 1982; Tremoulet and Feldman, 2000). Each of these features shares the property of self-propulsion, velocity changes that are initiated without physical contact, which manifests perceptually as a property of an animate creature. Stewart (1982) describes this core factor as motion that violates Newtonian laws, which is described by Scholl and Tremoulet (2000) as “hidden energy" possessed by animate bodies. In contrast, if an object travels in a consistent direction with sustained movement, or changes direction as a consequence of contact with another object, observers are typically not left with the impression of animacy (Stewart, 1982).

It is important to note that a single object on a featureless background was used in Tremoulet and Feldman (2000), which shows that animacy does not require the presence of other entities. Even with a single object, stimulus changes that are self-induced—and therefore consistent with a hidden energy—can trigger the attribution of animacy. Tremoulet and Feldman (2000) showed that when a short line segment travels along a straight line and changes direction without realigning its orientation to its new path, it is less likely to be perceived as animate compared to when it does realign. The same researchers demonstrated that circles, or more generally non-pointed shapes, are similarly perceived as less animate than shapes that are able to exhibit rotations, even if they traverse the same trajectory.

Much in the same way that eye gaze signals the intentional state of others, Gao et al. (2010) demonstrated the power of oriented features in shapes to convey complex mental states such as predatory desires. In these “wolfpack" demonstrations, arrows oriented toward a target are perceived as having intent directed at the target (as in wolves toward a sheep), even when the movements of the objects themselves were completely random. Computational modeling indicates that the attributions adults make when viewing these chasing animations reflects super-additive gains from the integration of high-level attentive tracking with salient perceptual cues for animacy (Gao et al., 2019). In the coming section we will discuss how an object, after showing cues of animacy, can behave in meaningful social ways.

## 3. Toward attributions of social behavior

An animate object can interact with the environment, for instance by wandering around another animate object, at varying levels of complexity. It has been debated whether understanding social interactivity requires high-level reasoning. Shu et al. (2018) addressed this question with decontextualized stimuli from real-life aerial videos of moving people. Observers more often rated the dynamic, decontextualized scenes as interactive rather than non-interactive or unsure, indicating there are critical visual motion cues between items that give rise to the perception of interactivity. Consistent with that hypothesis, the authors developed a computational model that lacked explicit high-level intentions and goals that nonetheless accurately predicted human judgements. This finding is consistent with the notion of directedness of interactions in driving perception in simple animations.

In contrast, Rasmussen and Jiang (2019) maintained that both low-level motion characteristics *and* high-level reasoning contribute to people's judgements of social interaction in Heider-Simmel animations. They based this conclusion, in part, on the observation that perceived interactivity differs when viewing the vignettes in forward vs. reverse. The ability to capture the influence of higher-order inferences in the forward-played movies, which was weaker when viewed in reverse, indicates an important factor of extended time-dependent, narrative-like contextual cues present in Heider-Simmel animations. Confirmation that more elaborate narratives are associated with more abstract inferences also comes from computational work in which models that incorporate contextual information in addition to object trajectory cues better fit measures of human action recognition (Roemmele et al., 2016).

Simple shapes can also elicit more complex attributions about thematic content of events, and the animations themselves may evoke emotional states in the viewers. When asked to categorize the narratives depicted by simple 3-dimensional animations of moving objects into film genres, people can consistently do so, identifying themes of non-fiction, comedy, drama, and action (Visch and Tan, 2009). Observers also report experiencing sympathy and rooting, for example toward struggling circles—or “underdogs"—that move uphill (Kim et al., 2008).

Because Heider-Simmel animations have the potential to engage more complex mentalizing, these movies have also been considered for use in assessing social intelligence, as an alternative to traditional written tests (Brown et al., 2022). This is particularly valuable to studies of cognitive development, in which children do not yet have the ability to read narratives. This also makes the study of social inferences derived from Heider-Simmel animations particularly valuable for comparative study of theory of mind abilities in non-human species. In the following section we discuss the developmental and comparative evidence for mentalizing abilities in children, non-human primates, monkeys and chicks. In the second half of the review, we will discuss neural evidence to support the behavioral findings.

## 4. Attributions of animacy, goals, and beliefs: a developmental approach

Babies are born with preferentially looking patterns directed toward socially meaningful features, including faces (Morton and Johnson, 1991; Buiatti et al., 2019), the eyes (Farroni et al., 2002), direction of gaze (Batki et al., 2000), biological motion (Simion et al., 2008), and animated shapes that move in accordance with cues for animacy, such as self-propelled motion and speed changes (Di Giorgio et al., 2017, 2021). Orienting toward simple shapes that convey animacy is apparent after only a couple of days of birth. This very early social orienting system is believed to reflect the function of a subcortical and more rudimentary orienting system at birth, which is subject to refinement over the next few years (i.e., Di Giorgio et al., 2016).

### 4.1. Attributions of goals

In parallel to the development of social cognitive systems, infants also possess an intuitive physics (Hespos and vanMarle, 2012) which after 2 months enables them to understand the basic properties of objects, such as solidity, cohesion, and invariance in object size, shape, pattern and color (Baillargeon, 2008). For example, continuity gives infants the expectation that a moving ball will stop when it comes in contact with a wall, and infants will look significantly longer in surprise if the ball passes through the wall (Spelke et al., 1992). With the development of an intuitive physics children understand the physical interactions between non-agent entities. At the same time an intuitive psychology helps infants understand the behaviors of agents. Around the age of 2 months, infants begin to react differently—through smiles and vocalizations—to the facial movements of people vs. to the facial movements of a doll (Legerstee et al., 1987), and by 5 months infants' looking patterns are consistent with attributing goals to the movements of human hands (Woodward, 1998).

Infants can also make goal attributions solely based on variability of behavior, without explicit cues for animacy. For example, when viewing a self-propelled box persistently moving toward a cone, 3-month-olds identify the cone as the goal of the box and will show heightened interest if the box approaches a newly introduced object (Luo, 2011). Around the same age, children can discern the social goals of a simple shape as it facilitates or impedes another shape's goals (Hamlin et al., 2010), and 3 months later show a preference toward the helper as compared to the hinderer (Hamlin et al., 2007). Older studies have also argued for children's ability to perceive goals in Heider-Simmel style animations, although those findings suggested later onset of this competence, at 6.5 (Csibra, 2008) and 9 months (Csibra et al., 1999).

**Figure 2:**
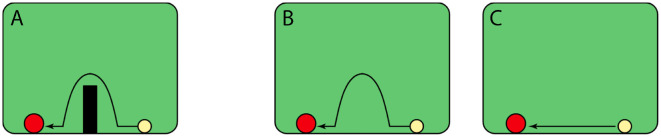
Rational goal-directed behavior of a shape moving towards the other by avoiding a barrier (familiarization) **(A)**. **(B, C)** Depict test trials where in the absent of the barrier, the animate object either travels along the same but now inefficient trajectory, or on a straight path to the goal, as expected from a rational teleological stance. Frames adapted with permission from redrawings of Gergely et al. (1995) by Gergely and Csibra (2003).

As proposed by Csibra et al. (1999), infants younger than one year are confined to a *teleological* stance by which they see phenomena in terms of purposes. In a review by Saxe et al. (2004) and inspired by Flavell (1988), this stance is described as making direct *connections* between objects. Importantly, in the teleological framework, infants utilize the psychological principle of rational action to understand goal-directed behavior such that an actor will approach its goal through the most efficient means as imposed by the physical environment [“situational" constraints; Gergely and Csibra (2003)]. Figure 2 shows examples of rational and irrational actions as studied by Gergely et al., 1995, with one shape reaching a goal either in presence of a wall or in its absence.

Constraints on rational actions can include the hidden beliefs of agents, which are not directly observable but nonetheless have the potential to guide more complicated actions that may otherwise be perceived as irrational. It is with the development of the *mentalistic* stance that more sophisticated mental reasoning is constructed, which allows the interpretations of more complex actions. This stance is termed as *representations* in Saxe et al. (2004)'s view or subjective experiences attributed to others. Gergely and Csibra (2003) and Saxe et al. (2004) agree in that the teleological stance/connections precede the mentalistic stance/representations in the course of development. They, however, differ in what they regard as “mentalistic". Gergely and Csibra (2003) believe that mental state attributions emerge only after the teleological stance, while Saxe et al. (2004) consider both connections and representations to be mentalistic. We will continue our discussion using the teleological/mentalistic model as it offers a less ambiguous framework.

### 4.2. Attributions of beliefs

Later in development children are able to attribute mental states to agents and understand that they hold subjective experiences of their own, which in turn enables the child to reason about complex actions driven by beliefs (Gergely and Csibra, 2003). Compared to the teleological stance with its components of goals, physical constraints, and actions, this more sophisticated mentalistic stance includes desires, beliefs, and intentions. Moreover, these internal states are interwoven such that desires define goals, beliefs shape implied constraints, and intentions lie behind actions. For example, in a study by Berry and Springer (1993) using motion pictures similar to the original Heider-Simmel animation, a 3-year-old girl reported the following description: “The daddy is chasing the little one around the house. He'll catch him. Well, he didn't catch him, so he got mad and broke the house and that's the end".

As proposed by Gergely and Csibra (2003), the mentalistic stance is also guided by the principle that agents will strive to achieve their desires through the most efficient means.^1^ One can therefore make inferences about intentions through mentalistic reasoning when observing behaviors, when desires and beliefs are known. Indeed, given any pair of the triple components of mental states, a prediction on the unknown one can be made.

It was traditionally believed that the mentalizing aspect of theory of mind, and in particular false belief representations, develops around the age of 3.5 (Wellman et al., 2001). However, it has more recently been argued that younger toddlers also possess an understanding of false beliefs. This was demonstrated in a help task experiment by Buttelmann et al. (2009), in which 18-month-olds observed an actor placing a toy under one of two boxes. Another actor then moved the toy to the other box, either in presence of the first actor (true belief condition) or in the actor's absence (false belief condition). In both conditions, the first actor subsequently reached for the empty box. Whereas in the true belief condition the young toddlers helped the actor open the empty box, in the false belief condition the toddlers guided the actor to the correct box. This implies the belief that the actor was seeking the toy but held a false belief about its location. This main effect of underlying belief was demonstrated in another study using the same paradigm in 15-month-olds and measuring looking time and violation of expectation (Onishi and Baillargeon, 2005). Toddlers in this experiment looked longer when the actor returned and reached for the box where the toy actually hid, showing that 15-month-olds expected the actor to choose the box based on her false belief.

There is further evidence that children reason about the internal states of others' minds very early after the first birthday (Surian et al., 2007). The researchers measured the looking time of 13-month-olds as they watched animations of an agent looking for food and found longer fixations toward actions that violated false beliefs. Interestingly, however, a caterpillar played the main role in the animations rather than a human actor, indicating that children's ascription of complex actions to minds is not restricted to humans. Therefore, similar to animacy and goal attributions, higher level attributions of mental states can also occur toward a variety of objects.

More recently, evidence shows that even around 10 months children are capable of representing mental states of others to distinguish between pro- and anti-social behaviors (Hamlin et al., 2013). In a social evaluation task, children observed a puppet show in which a lion showed a preference toward one of two objects, either in the presence or in the absence of two elephants. The elephants then lifted doors to give the lion access to an object. Children preferred the prosocial agent (the elephant that lifted the door to the preferred toy) only when the elephants had seen the lion's initial preference. Otherwise, if the elephants were not present to see the lion's preference, children did not evaluate their subsequent door lifting as pro- or anti-social, and chose one elephant randomly. The 10-month-olds therefore showed preference based on the match between the implied desires and actions of the puppets, showing some understanding of the mental states of others.

Hamlin et al. (2013)'s finding brings theory of mind to the first year of life, although it is not the earliest evidence to do so. We will discuss in the coming section how causality studies might have suggested even an earlier age for the emergence of theory of mind, possibly around 8–10 months (Rochat et al., 2004). As we touched on earlier, it is important to note that these earlier findings of theory of mind at 13 (Surian et al., 2007), 10 (Hamlin et al., 2013), and 8–10 months— discussed next—all involve non-human protagonists, i.e., an animated caterpillar, animal puppets, and circles. Whether or not attributing mental states to simpler agents develops earlier compared to attributions toward humans requires further investigation (Carey, 1985). Perceiving goals, however, has been shown to occur earlier with simple geometric shapes (Hamlin et al., 2010; Luo, 2011) than with a human hand (Woodward, 1998) as reviewed in the previous section.

### 4.3. Inferred physical and social causality

In this section, we discuss interactions between objects and focus on events that involve causal inference, as well as on factors that break causal links. Drawing on the teleological-mentalistic framework, we investigate causality as it occurs either within physical constraints (i.e., physical causality) or according to mental states (i.e., social causality). Note that causality can be studied in various forms, for example as sunshine causes a plant to grow, but our focus remains on animacy and proximal and immediate interactions.

We previously discussed how an object is perceived as animate and moves purposefully, which are important first steps in studying causality. Consider an event with circle A moving from rest on a straight path toward a second circle, B. As A hits B, B starts moving on about the same direction, resulting in a causal interaction. This is a case of physical causality and specifically an example of a launching effect, as illustrated in the classic works of Michotte (1963) on perceptual causality. Importantly, the link between the two objects will break if they violate physical laws, for instance if they leave temporal or spatial gaps between them. That is to say, in our launching example, if B begins moving not immediately after the moment of impact, or if there is distance between the stopping point of A and starting point of B, then A is not perceived as physically causing the movement of B. Under these circumstances, the principle of rationality would be unable to explain the event in teleological terms.

What do developmental studies teach us about the attribution of causation by contact and causation at a distance? Would infants perceive social causality if causation occurs at a distance? Spelke et al. (1996) suggested that 6-month-olds might understand that people can interact without contact. Below we review two studies that test this hypothesis on infants under age 1, with stimuli of simple shapes.

Schlottmann and Surian (1999) showed 9-month-olds launching events with two squares, with one moving toward the other and stopping at a distance before the second square moved. Interestingly, and contrary to Michotte (1963)'s predictions, infants derived an impression of causality despite no contact between the shapes. The causal chain, in this situation, did not break with a spatial gap. It is possible that younger children understand causality within physical constraints, and later around 9 months develop an understanding of social causality. Rochat et al. (2004) tested this by directly comparing inferred causality across ages, in an experiment with animated displays of two chasing discs. While never making contact, the chaser moved at a slow but steady pace toward the chasee which accelerated away, and it was programmed to move constantly closer to the chasee without following its path. Hence, the chaser sought the chasee's heat rather than directly following it. This heat-seeking behavior is an essential attribute of the discs in this experiment, because it renders improbable any direct physical connection between them. Three to 10-month-olds participated in this study, but only infants between 8 and 10 months tended to dishabituate to a role reversal between the chaser and the chasee, which shows their understanding of social causation.

As Rochat et al. (2004) stated, “action at a distance is a trademark of social exchanges", and it is around 8–10 months of age that children make a transition into understanding such mentalistic interactions. Before this age, between 3 (Luo, 2011) and 8 months, infants' thinking about others is limited under a teleological stance. Tomasello (2001) had indeed described a transition in social-cognitive development around 9 months (also coined as the “9 month revolution") when children come to understand others as intentional agents, similar to the perceptual-cognitive change reviewed here. In search for the evolutionary origins of this transition, in the coming section we will review studies of goal-directedness and theory of mind in primates, and will then delve into evidence from neuroscience regarding when and how a mind-understanding mind is developed.

## 5. Attributions of goals and beliefs by monkeys and apes

Humans have tended to consider theory of mind a distinctive human capacity, but numerous discoveries in primates suggest that this notion may be a myth. Bonobos and chimpanzees, who diverged from humans about 7 million years ago, are examples of Great Apes who exhibit the ability to attribute animacy to abstract shapes and an understanding of goal-directed behavior. In an experimental design inspired by Hamlin et al. (2010), Krupenye and Hare (2018) showed bonobos animations of two simple shapes engaged in apparent helping or hindering interactions, with an added cue for animacy (eyes) attached to the shapes. Whereas 3-month-old human infants gaze preferentially to prosocial agents, the bonobos' preference was for the hinderer. This finding conforms with bonobos' behavior in real-world scenarios as they choose dominant individuals over subordinates.

Evidence shows that chimpanzees can also attribute goals to objects and this teleological representation is bounded by the principle of rationality. Uller (2004) measured eye gaze in infant chimpanzees using a task first developed by Gergely et al. (1995) for humans (as illustrated in Figure 2). In this task the chimpanzees were familiarized with an animation of a rectangle traveling along a parabolic path to avoid a barrier and reach a circle. In the test phase without the barrier, the chimpanzees then observed the triangle moving either along the same parabolic trajectory (test 1), or on a straight line (test 2). The chimpanzees looked longer at test 1 that depicted the inefficient, irrational parabolic path, evidence that they recognized the goal and also expected the triangle to move straight toward it.

**Figure 3:**
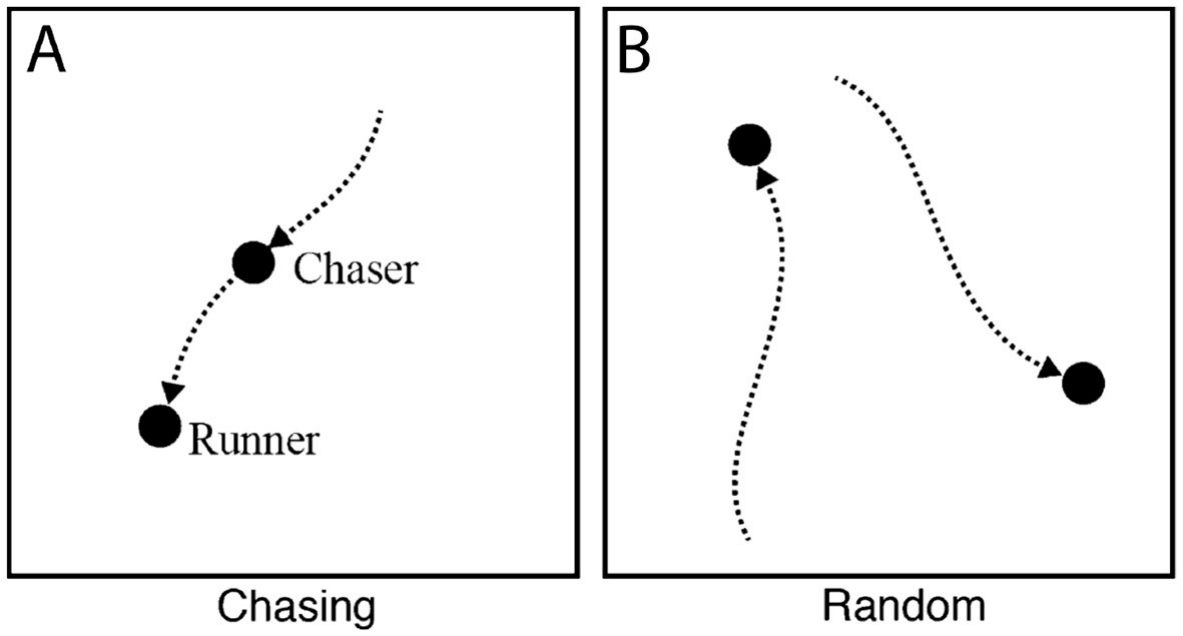
Chasing **(A)** and random **(B)** animations showing goal-directed path following and undirected behavior, as shown to macaques and squirrel monkeys in the studies of Atsumi et al. (2017) and Atsumi and Nagasaka (2015). Both groups of monkeys exhibited an understanding of directedness by attributing goals to the chaser. Adapted with permission.

Old World monkeys diverged from the Great Apes about 20–30 million years ago (Wood et al., 2007; Hayashi et al., 2020). The evidence for whether monkeys can also reason about goal-directed behavior is more mixed. In an experiment with Japanese macaques (*Macaca fuscata*, who belong to the Old World family), monkeys were shown animations with two discs that either moved randomly (Figure 3B) or depicted a runner that moved randomly with a chaser that pursued the same trajectory (chasing, Figure 3A; Atsumi et al., 2017). Similar to human observers who were also included in this study, macaques successfully recognized and selected the chasing events to earn food rewards, which is argued to be evidence that monkeys understand goal-directed behavior. Because the monkeys earned food for their selection, however, others have criticized the study as instead reflecting learned associations between certain low-level movement characteristics and reward (Schafroth et al., 2021).

Acknowledging the issue of learned associations, Schafroth et al. (2021) investigated theory of mind capacities of rhesus monkeys (*Macaca mulatta*, also belonging to the Old World group) in a free-viewing paradigm. This experiment used the same classic Heider-Simmel animations as in human studies, which allows for better interspecies comparisons even though such movies might not be ethologically relevant to monkeys. Whereas humans have longer fixation durations (an indicator of deeper processing) when viewing sequences of interactions best understood using a mentalistic stance, the monkeys fixated longest on animations that could be interpreted from a teleological (goal-directed) stance. Importantly, however, this effect vanished when perceptual variables, including peak motion and motion variability were included as covariates. The authors therefore concluded that there is no evidence that rhesus monkeys have an understanding of goals from simple shapes. They also noted that the monkeys were largely disinterested in the more complex theory of mind animations and glanced around the testing room during those events.

Monkeys' disengagement from Heider-Simmel stimuli might be due to the abstract symbolic nature of these animations. Indeed, in an experiment with rhesus monkeys who observed a human actor reaching for food hidden in one of two containers, the monkeys looked preferentially at the actor's target, evidence that they can make inferences about goals (Wood et al., 2007). Interestingly, this preference was evident only when the action was performed rationally. The monkeys gazed preferentially at the target when actor's hands were occupied holding another object and he reached for the container with his elbow, but not when the actor had empty hands and still (inefficiently) used his elbow. This indicates that the rhesus monkeys were sensitive to the rational nature of the action, consistent with adopting a teleological stance as taken by bonobos (Krupenye and Hare, 2018). Other primates including chimpanzees and tamarins (New World monkeys) were also tested in this study and showed similar rational teleological stances.

New World monkeys diverged from Old World monkeys and Apes about 30–40 million years ago (Wood et al., 2007; Hayashi et al., 2020). The goal-understanding of New World monkeys has been tested with simple animations as well, but with less promising results. Atsumi and Nagasaka (2015), for instance, found squirrel monkeys to be capable of perceiving the chasing of discs, using a similar design as in Atsumi et al. (2017). The same issue of over-training and reward associations, however, also applies here. Another study with marmoset monkeys (*Callithrix jacchus*; Burkart et al., 2012) has also shown that New World monkeys are incapable of attributing goals to moving objects such as a box, but can do so when observing a conspecific, similar to Wood et al. (2007)'s findings with monkeys observing human actors.

There is reason to believe that both Great Apes and Old World monkeys are capable of more sophisticated reasoning about actions than allowed by teleological representations. In a study conducted by Kano et al. (2019) Great Apes (including bonobos, chimpanzees, and orangutans) watched an ape-like actor who hid an object under one of two boxes in the presence of a human-like actor, and then moved the object to the other box when the second actor was away. After the return of the human actor, Great Apes preferentially fixated at the first box, indicating an expectation based on the actor's knowledge and guided by his false belief. A similar paradigm was used in an experiment with Japanese macaques (Hayashi et al., 2020), who also looked longer at the box where they expected the actor to falsely believe to be the location of a hidden object. Furthermore, by disrupting the medial prefrontal cortex (mPFC) of the macaques (by injections of an inhibitory drug) and consequently eliminating the animals' anticipatory looking toward the false-belief location, the authors suggested a causal role for this brain region in mentalizing. This disruption left macaques' other abilities including movement tracking intact.

In sum, a teleological understanding of the world might date back to New World monkeys, about 40 million years ago. More cognitive mental state attributions are however more evolutionary recent, as discovered with false-belief tasks in Old World monkeys who share 30 million-year-old ancestors with Apes. Findings specifically with Heider-Simmel animations were less reliable. It is possible that only Great Apes are capable of engaging with symbolic representations of social behavior, and that is also restricted to attributions of goals.

## 6. The neural underpinnings of social cognition

Numerous studies have linked brain areas involved in social cognition to perceived animacy from animations of simple shapes. Using fMRI, Gobbini et al. (2007) investigated neural responses of human adults to animations involving rigid social interactions that conveyed goal-directed action, and to false belief stories. Interestingly, and consistent with previously reviewed behavioral reports, two distinct systems were evoked by goal-directed animations and mentalistic stories. These systems were widely distributed, but notably involved the posterior superior temporal sulcus (pSTS) for representations of goals, and the temporo-parietal junction (TPJ) for mental state attributions, areas known as part of the neural system for theory of mind. Both the pSTS and the TPJ were also found together in the PET scans of individuals in another study who watched mentalistic Heider-Simmel like animations vs. simple action animations that conveyed no social meaning (Castelli et al., 2000). The authors also reported the involvement of the medial prefrontal cortex (mPFC), a midline structure associated with introspective thought, when viewing animations depicting mentalistic attributions. Martin and Weisberg (2003) found further evidence that social interactions between shapes engage the social cognitive brain network. Using long narrative vignettes (21 s) of simple geometric shapes that depicted either social interactions or mechanical relations, the researchers identified distributed patterns of neural activity bilaterally on the STS and within ventral parts of the mPFC (vmPFC), the latter finding proposed to be the results of the narrative eliciting emotional attributions.

These neural findings in adults have identified a set of brain systems that are widely accepted as the so-called “social brain". How does this social brain system develop in infants when the cognitive processes that support perceived animacy and social cognition are coming online? It is very challenging to engage young children in task-related experiments, and maybe near impossible for infants and neonates, especially when conducting neurophysiological measures. While engaging children with specific tasks remains unlikely, resting-state task-free paradigms are starting to elucidate the development of brain networks. The most common of these paradigms include imaging during natural sleep which has been widely utilized for younger children, i.e., newborns (Fransson et al., 2009), children under 3 years (Howell et al., 2019), and 2–4-year-olds (Redcay et al., 2007), and during wake with passive watching of movies of their choice for older children, as conducted by Howell et al. (2019) on children above 3 years, and by Emerson et al. (2015) on 6-year-olds. Although the latter approach can still shed light on brain development, it comes with the issue of engaging children with visual and auditory stimuli, which has potential to shift cortical networks into task-driven states rather than being structured by intrinsic connectivity (Biswal et al., 1995).

Because of our particular interest in the first two years of life in this review, our focus will be on naturally sleeping children, in which spontaneous, low-frequency neural activity results in the emergence of intrinsic functional networks of the brain, known as resting-state networks (RSNs). Of the different modalities used to investigate the correlation between brain regions, resting-state functional MRI (rs-fMRI) has shown more promise as it comes with higher spatial resolution, although the importance of integration with high temporal resolution techniques, including EEG and MEG, is worth noting (Grayson and Fair, 2017). We review resting-state networks—specifically the default mode network—in adults next, as a framework for the section that follows on the development of resting state.

### 6.1. Resting-state and social cognitive networks in adults

In the resting state, adult brain networks organize into a relatively small number of consistent states, which include the default-mode network (DMN), a vastly distributed network consisting of regions including but not limited to the inferior parietal cortex (the inferior parietal lobule, IPL; and the temporo-parietal junction, TPJ), the posterior cingulate cortex (PCC), and the mPFC. This network is the one most commonly identified in the absence of external stimuli (Buckner et al., 2008) during which individuals engage in stimulus-independent or spontaneous thought that may consist of dreaming, mind-wandering or creative thinking (Christoff et al., 2016). The cognitive processes associated with the DMN are commonly linked to internally directed thought, which has been shown to include memory retrieval, planning for the future, and reasoning about others (Harrison et al., 2008), all key cognitive functions for developing mental models of situational context that facilitates navigating social interactions (Yeshurun et al., 2021). Evidence shows that at the core of such functions lies an understanding of the self, which can send projections to these processes or act as a reference (Buckner and Carroll, 2007; Buckner et al., 2008).

**Figure 4:**
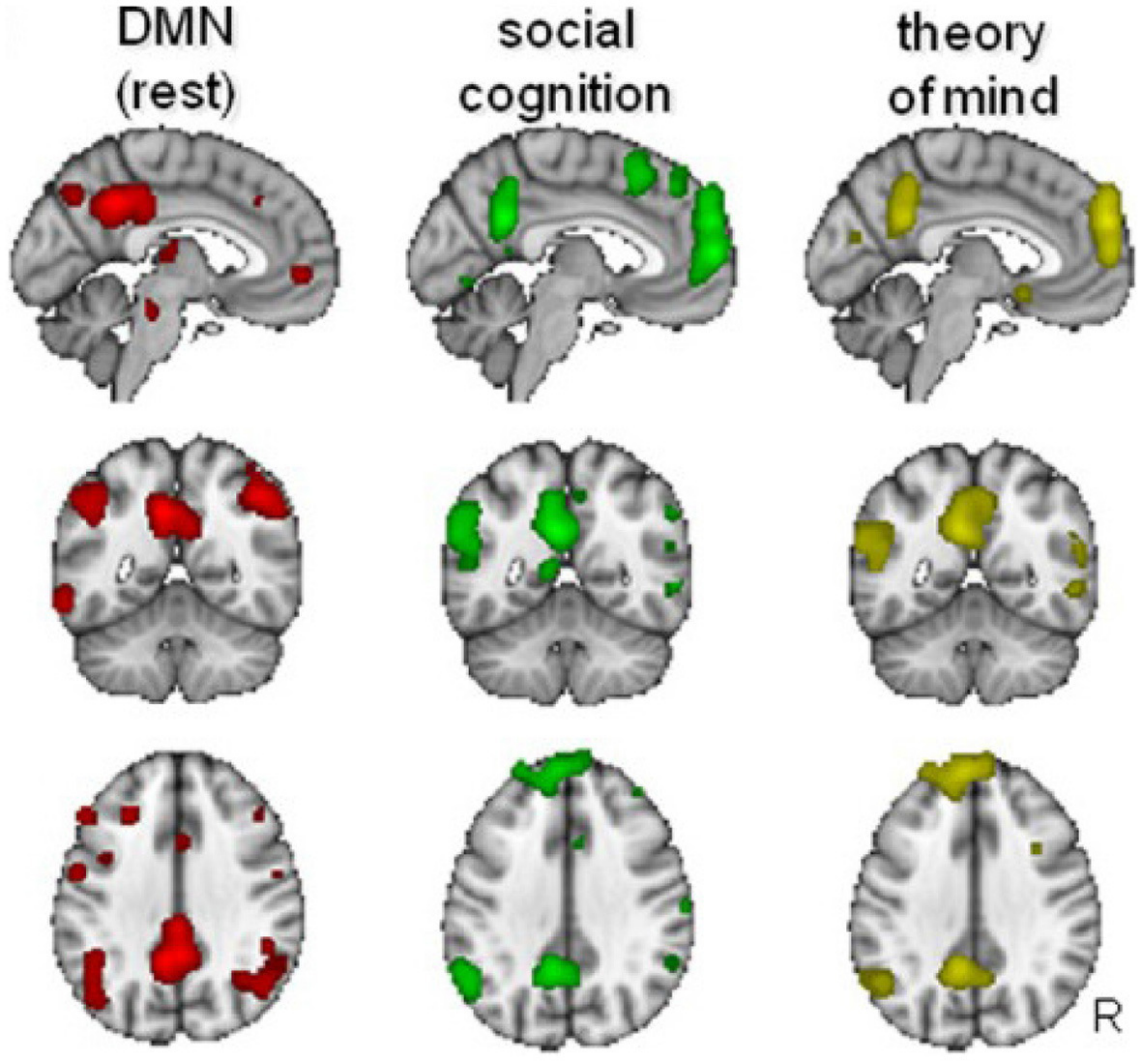
Overlap between the default-mode network (DMN), social cognition, and theory of mind. Similarities exist at the posterior cingulate cortex (PCC), the medial prefrontal cortex (mPFC), and the temporo-parietal junction (TPJ). Adapted from Mars et al. (2012) (CC-BY-NC 3.0).

Figure 4 illustrates the similarities between the DMN, the social cognitive network, and the system involved in theory of mind, with clear overlap in the parietal, posterior medial and medial frontal regions. The IPL is involved both in the DMN and when humans think about themselves vs. others (Vogeley and Fink, 2003; Schilbach et al., 2008; Mars et al., 2012). Posterior medial parts of the DMN which include the PCC and precuneus are also involved in social cognitive processes such as mentalizing (Saxe and Powell, 2006) and social interactions (Schilbach et al., 2006). These complex systems also have subdivisions within. For example, the TPJ can be split into a posterior and anterior region, which are known to play key roles, respectively, in mentalizing and orientation of attention (Patel et al., 2019). The DMN most strongly overlaps with the posterior TPJ (Mars et al., 2012), which has been associated specifically with the attribution of intentions (Atique et al., 2011).

**Figure 5:**
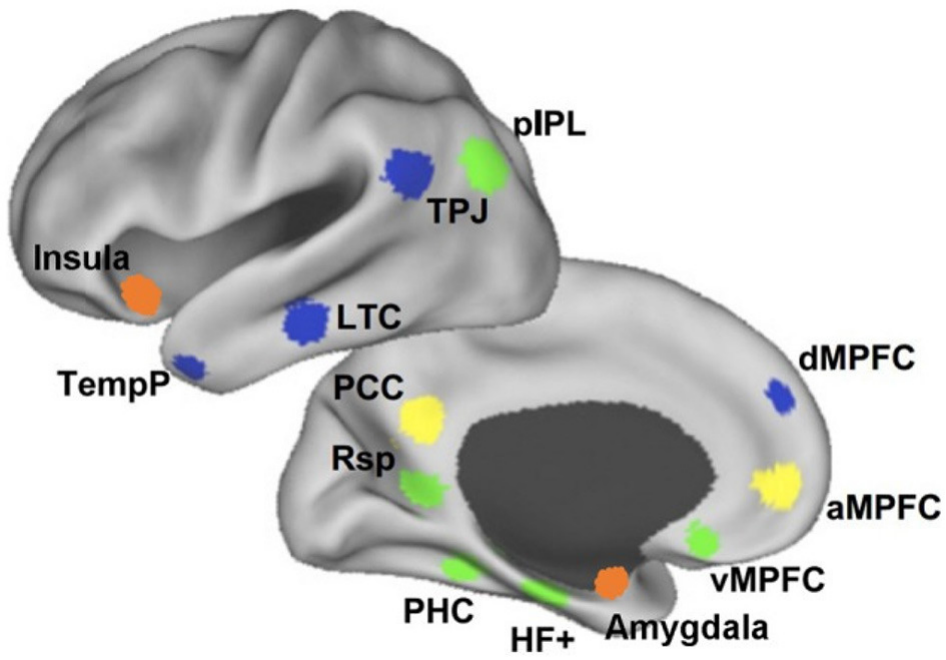
Subdivisions of the DMN. The vmPFC (green), through connections with the MTL and the IPL, represents self-relevant thought. The amPFC (yellow) connects to the PCC to draw distinctions between the self and others. The dmPFC (blue) is involved in reasoning about others together with the TPJ. Adapted from Li et al. (2014) (CC-BY 3.0).

The mentalizing role of the TPJ engages a widely distributed network that includes the mPFC (Mason et al., 2008; Burnett and Blakemore, 2009; Atique et al., 2011; Baumgartner et al., 2012; Hervé et al., 2012). This network is implicated in mentalizing particularly when reasoning about hidden beliefs that are internal to the mind (Lieberman, 2007). The mPFC has been divided into three subdivisions at its ventral (vmPFC), anterior (amPFC), and dorsal (dmPFC) sides, each with distinct functional specialization and associated network (see Figure 5). Whereas the dmPFC is most activated when selectively reasoning about others (Dd et al., 2012; Denny et al., 2012), the vmPFC involves self-relevant representations and the amPFC is engaged during tasks that require drawing distinctions between self and others (D'Argembeau et al., 2005; Heatherton et al., 2006; Andrews-Hanna et al., 2010). The transition from understanding the self in ventral parts of the mPFC, to representation of others in the dmPFC is demonstrated by Li et al. (2014) as well. Together, the TPJ and mPFC have been linked to the consolidation of recently learned social information, as demonstrated by increased connectivity when measured after exposure and during rest (Meyer et al., 2019).

It is important to note that assignment of reward to social signals has also been found within the social network (Frith, 2007). The TPJ, for instance, shows increased functional connectivity with reward processing regions during empathy (Janowski et al., 2013) and charitable donations (Hare et al., 2010), which can be described as social motivation being associated with social reward (Grimm et al., 2021); more generally, the role of the TPJ might be to compare predictions about the environment with actual outcomes (Abrahamse and Silvetti, 2016). Social reward is mainly processed at the mesolimbic system, which includes the ventral tegmental area (VTA), the PFC, and striatum (Meshi et al., 2013; Serafini et al., 2020), although these regions are involved in various non-social processes as well. The vmPFC is also associated with reward and punishment, and through connections with the amygdala and insula (Carmichael and Price, 1995; Akitsuki and Decety, 2009; Otti et al., 2010), involves in perceiving emotions in social contexts. Social reward, punishment, and motivation have been found in the dmPFC as well (Fehr and Camerer, 2007; Kohls et al., 2013). It is proposed that the vmPFC is involved in self-referenced reward (Dang et al., 2019), while the dmPFC that is linked to cognitive tasks, processes reward information related to others (Apps et al., 2012; Lockwood et al., 2015).

It is worth mentioning that the activation of the social cognitive network is also influenced by cueing and attention, specifically with Heider-Simmel style animations. In an fMRI study, Tavares et al. (2008) showed significant boosts in the social brain network when selective attention was paid to social meaning vs. to spatial properties of the movies. Participants were cued either by the word “behavioral" or by “spatial" before observing animations that showed two circles (i.e., agents) moving through constraints. In the spatial condition, participants were asked to attend to motion features such as speed or trajectory patterns. When cued with “behavioral", however, they were instructed to identify the type of interaction between the circles. Cueing can therefore enhance attributions of mental states toward movies with simple shapes. The pSTS, in particular, has however been shown to respond to interactivity cues irrespective of task, suggesting its automatic involvement in detecting animacy (Schultz et al., 2005).

We will review resting-state development in the coming section, and will also further discuss the roles of the TPJ and mPFC in theory of mind. First, though, it is worth noting that within the DMN various hubs have been identified, which form the basis for the developmental trajectory of the system as a whole. Both Mars et al. (2012) and Buckner et al. (2008) have identified the PCC-Rsp and the mPFC as DMN hubs when evaluated in the resting-state, with the potential for hub properties in the parietal regions of the DMN based on task-based studies of social cognition (Yang et al., 2015; Patel et al., 2019). The PCC, in particular, plays an important role in DMN development (Gao et al., 2009), as will be discussed below. This region not only acts as a key hub within the DMN, it is also involved in attributions of mental states. As Lombardo et al. (2010) have shown, the PCC functionally connects to the TPJ and the mPFC when mentalizing about the self and others, and also responds to self-relevant emotional events (Vogt et al., 2006).

### 6.2. Resting-state in children

As discussed earlier, resting-state fMRI has been a common approach for studying the functional networks of the young brain. In a study of cortical network activity in the first two years of life, Gao et al. (2015b) identified nine functional networks, which divide into topologically adult-like primary networks and widely distributed higher-order networks that are incomplete in younger children. The former includes an early visual and a sensorimotor network, while the latter consists of multiple networks, including a DMN, that become more consolidated through childhood and adolescence (Fair et al., 2008; Mak et al., 2017). All higher-order networks appear in forms that are rudimentary as compared to their adult version.

**Figure 6:**
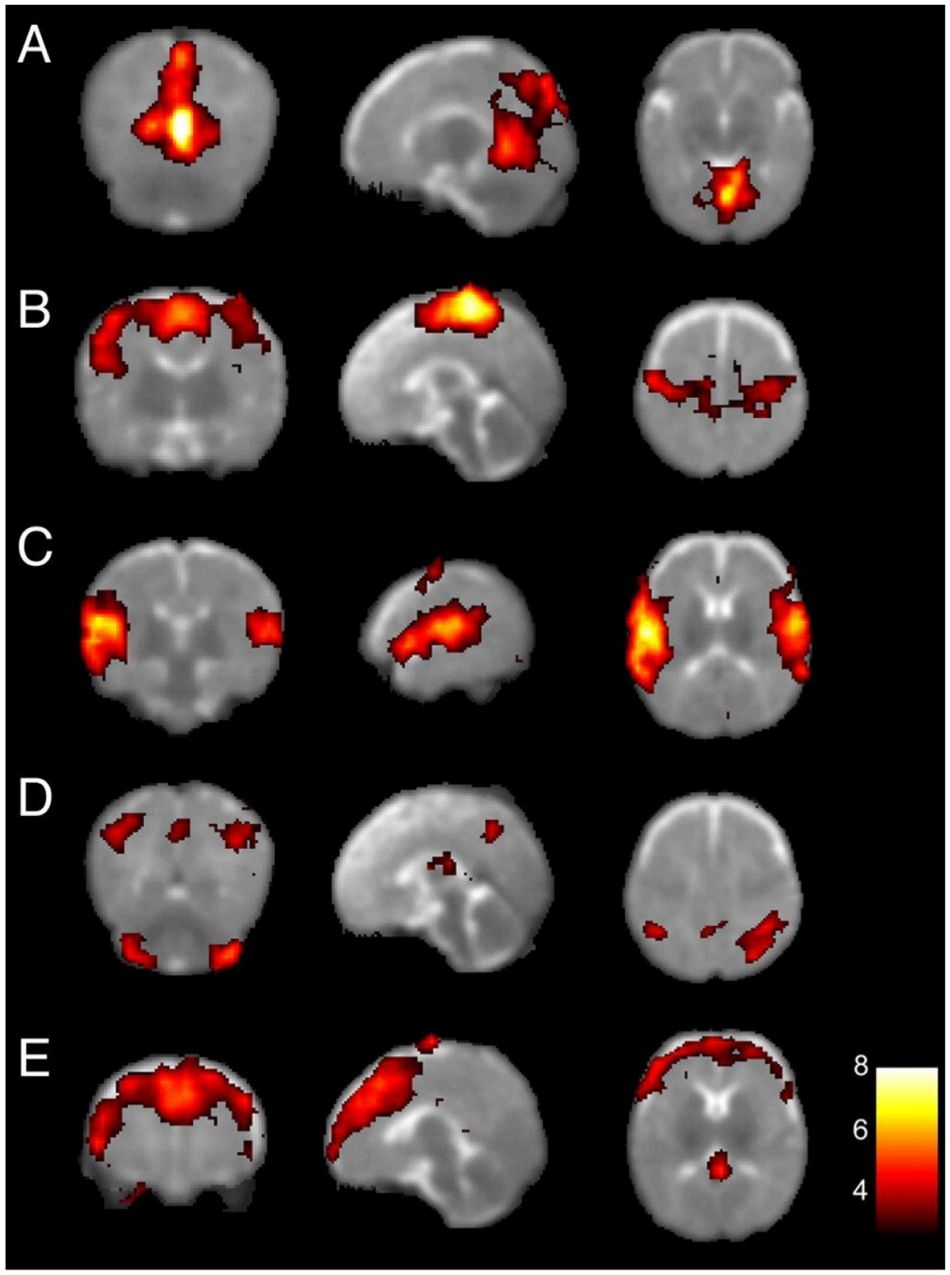
Resting-state networks at birth. Primary visual **(A)**, bilateral sensorimotor **(B)**, bilateral auditory **(C)**, proto-DMN, consisting of the lateral parts of the cerebellum, the posterior mid-parietal areas including the precuneus, and the posterior lateral parietal cortex **(D)**, and prefrontal **(E)** networks. Adapted from Fransson et al. (2007). Copyright (2007) National Academy of Sciences.

Investigations on preterm infants at term-equivalent age have also shown the existence of five resting-state networks, as illustrated in Figure 6. The primary visual, auditory, and somato-motor networks resemble the adult counterparts and will only undergo fine developments later in the first 2 years of life (Lin et al., 2008; Gao et al., 2015b). The other networks take more time to develop into mature forms, although not directly into an adult equivalent. In particular, the medial and lateral parietal networks (shown as network D in Figure 6) are regarded by Fransson et al. (2007) as a proto-DMN, which includes the posterior parts of a well-formed DMN. More higher-order early networks have also been detected in preterm infants, including an executive control network (Doria et al., 2010).

Regions that form the proto-DMN, which are mainly bilateral, later develop into brain regions linked to mentalizing, consistent with behavioral findings of goal attribution in infants as young as 3 months (Luo, 2011). Connections within this network, as well as other infant networks, will drastically grow during the first compared to the second year, while between-network segregation also occurs (Gao et al., 2015b). Pruett et al. (2015) specifically demonstrated significant DMN development in the second half of the first year, by showing the network's involvement (together with the dorsal attention network) in identifying 6 vs. 12 month-olds based on their patterns of functional connectivity in the resting-state.

It takes over 10 years for the DMN to find its complete mature form (Hoff et al., 2013; Fan et al., 2021), although this time course will be delayed in autistic children who show greater modularization driven by reduced between-subnetwork connectivity (Bathelt and Geurts, 2021). As a milestone in DMN development, connections within the PCC strengthen around the age of 2 to turn it into a hub that functions to link the posterior and anterior regions of the DMN (Gao et al., 2009). This posterior-anterior growth is of chief importance here because of its possible relevance to teleological-mentalistic representations. Other developmental patterns, however, have been identified, in inferior to superior and medial to lateral directions (Gao et al., 2015a).

### 6.3. Social predisposition or learned competence?

If a social tendency, such as the sensitivity to self-propelled motion (Di Giorgio et al., 2017), is evident in newborns even only after a couple of days, it is important to ask whether such tendency is indeed innate or has been influenced by learned mechanisms. As highly altricial species, human babies cannot be deprived of early learning and might never be suitable for investigations of inborn biases. Precocial animals such as domestic chicks, however, can be kept in complete darkness after hatching until tested for predispositions, making them feasible models for nature vs. nurture research.

Similar to human newborns, newly hatched chicks with no prior visual experience are sensitive to face-like configurations (Rosa-Salva et al., 2010; Rosa Salva et al., 2011), biological motion (Rugani et al., 2015; Miura et al., 2020), and to simple shapes that show animacy through self-propelled motion (Mascalzoni et al., 2010), speed change (Rosa-Salva et al., 2016; Versace et al., 2019; Lorenzi et al., 2021), orientation to motion direction (Clara et al., 2009; Rosa-Salva et al., 2018), or gradual trajectory changes (Rosa-Salva et al., 2016). Rosa Salva et al. (2011) tested the preference toward faces in visually naïve 2-day-old chicks (*Gallus gallus*) who never saw the experimenter's face or the face of another chick. They found that chicks preferred human faces to frequency- and color-matched control scrambled images, just as newborn human infants do. The use of human faces for chicks is proposed to demonstrate an innate non-species specific face preference (Morton and Johnson, 1991) that gives way to species, breed and identity selective preferences through environmental exposure during sensitive periods of development (Rosa-Salva et al., 2021).

Newborn chicks also have a spontaneous preference for cues that signal animacy Rosa-Salva et al. (2016). Naïve domestic chicks were placed in a runway apparatus that displayed one shape moving at constant speed at one end, and a speed-changing shape at the other end. The latter accelerated at one third of its path and decelerated to its initial speed at two thirds of the trajectory. Chicks preferred and approached the second shape, showing a predisposition for the animacy cue of speed change. Interestingly, similar to the early transient non-species face preference in chicks, this predisposition exists for only 24 h after hatching, and fades two days later (Rosa-Salva et al., 2021), although it can be restored by administering a hormone associated with the opening of critical windows in imprinting, at least in female chicks (Lorenzi et al., 2021). It is also worth noting the importance of exposure to environmental cues for animacy, as occluding the speed changes will suppress the preference (Rosa-Salva et al., 2016). Predispositions for animacy can be diminished with embryonic injections of Valproic Acid (VPA) as well, which models the behavioral deficits observed in autism spectrum disorder (ASD). Sgadò et al. (2018) showed that VPA exposure impairs newly hatched chicks' predisposition for hen-like objects, while it leaves their subsequent learning intact, evident from their normal imprinting behavior toward a familiar simple shape.

Subcortical—specifically limbic—structures may play an important role in the early detection of animacy and in the imprinting behavior of vertebrates, which function prior to postnatal learning. The nucleus taeniae and arcopallium (amygdala homologues) together with the septal nuclei and the preoptic area (POA) of the hypothalamus of visually naïve chicks are linked to viewing live conspecifics (Mayer et al., 2017a,b) and hen-like objects (Mayer et al., 2019). The POA along with the septum have also been linked to viewing speed changes associated with animacy Lorenzi et al. (2017). The function of limbic structures in promoting perceived animacy may serve to support the imprinting process between newborn chicks and their early social partners given the role of these structures in emotional valence (O'Connell and Hofmann, 2011).

## 7. Conclusions

Like adults, human newborns show preferences toward animacy cues, and are able to connect animate objects to their goals as early as the age of 3 months. They then are capable of attributing beliefs to others toward the end of the first year and understand subjective minds. Here we suggest that the development from a proto-DMN into a maturing DMN between birth and the age of 2 supports the transition from perceiving goals to making attributions about mental states (Pruett et al., 2015). This is consistent with the dramatic development of social cognitive functions that emerge around 9 months of age, specifically with the behavioral findings of false beliefs before the age of two (Onishi and Baillargeon, 2005; Surian et al., 2007; Buttelmann et al., 2009) and even before the first birthday (Rochat et al., 2004; Hamlin et al., 2013). It is important to note that a fully mature DMN is not yet emergent when early theory of mind has appeared. Mind-understanding begins functioning before the first birthday alongside a primitive DMN that includes the TPJ, which represents externally-focused processes. Over time the maturation of the DMN includes the formation of a PCC hub and connectivity to the mPFC, consistent with the emergence of reasoning about internal states of others (Lieberman, 2007). Further investigation is however needed to find direct connections between the early DMN and social cognition.

Here we also reviewed findings of goal and belief attribution in monkeys and apes to discuss whether the teleological-mentalistic sequence found in humans has evolutionary origins. A teleological understanding of the world indeed emerges before mentalizing in evolutionary terms, as evident from findings of goal attribution in New World monkeys, and from findings of mentalizing in Old World monkeys who emerge from a more recent branch in evolution. With Heider-Simmel style animations, which represent social interactions through symbolic abstractions, only Great Apes show an engagement which is limited under a teleological stance. Recognition of animacy cues (e.g., speed change), more generally, has been found in visually naïve chicks as well, suggesting that a wide range of vertebrates are predisposed to animacy.

## Author contributions

All authors listed have made a substantial, direct, and intellectual contribution to the work and approved it for publication.

## Footnotes

^1^It should be noted that rationality as being discussed here pertains to mental models of agency rather than the theory of the rational decision-maker as proposed in other fields including behavioral economics. The traditional rational-agent model in economics, in particular, assumed that human decisions are rational. Prospect theory (Kahneman and Tversky, 1979), on the other hand, has argued that human preferences are often *frame-bound* as opposed to *reality-bound*, and deviations from reality toward subjective frames result in inconsistencies in behavior, and therefore irrationality.
